# A high-throughput phenotyping method for sugarcane rind penetrometer resistance and breaking force characterization by near-infrared spectroscopy

**DOI:** 10.1186/s13007-023-01076-0

**Published:** 2023-09-28

**Authors:** Yinjuan Shen, Muhammad Adnan, Fumin Ma, Liyuan Kong, Maoyao Wang, Fuhong Jiang, Qian Hu, Wei Yao, Yongfang Zhou, Muqing Zhang, Jiangfeng Huang

**Affiliations:** 1 State Key Laboratory for Conservation and Utilization of Subtropical Agro-Bioresources, Guangxi Key Laboratory of Sugarcane Biology, Province and Ministry Co-Sponsored Collaborative Innovation Center of Canesugar Industry, Academy of Sugarcane and Sugar Industry, College of Agriculture, Guangxi University, Nanning, 530004 Guangxi China; 2 Guangxi China-ASEAN Youth Industrial Park (Chongzuo Agricultural Hi-Tech Industry Demo Zone), Chongzuo, 532200 Guangxi China; 3 Nanning Sugar Industry Co., LTD, Nanning, 530028 Guangxi China

**Keywords:** Sugarcane, Mechanical strength, Rind penetrometer resistance, Breaking force, Phenotyping

## Abstract

**Background:**

Sugarcane (*Saccharum spp.*) is the core crop for sugar and bioethanol production over the world. A major problem in sugarcane production is stalk lodging due to weak mechanical strength. Rind penetrometer resistance (RPR) and breaking force are two kinds of regular parameters for mechanical strength characterization. However, due to the lack of efficient methods for determining RPR and breaking force in sugarcane, genetic approaches for improving these traits are generally limited. This study was designed to use near-infrared spectroscopy (NIRS) calibration assay to accurately assess mechanical strength on a high-throughput basis for the first time.

**Results:**

Based on well-established laboratory measurements of sugarcane stalk internodes collected in the years 2019 and 2020, considerable variations in RPR and breaking force were observed in the stalk internodes. Following a standard NIRS calibration process, two online models were obtained with a high coefficient of determination (*R*^*2*^) and the ratio of prediction to deviation (RPD) values during calibration, internal cross-validation, and external validation. Remarkably, the equation for RPR exhibited *R*^*2*^ and RPD values as high as 0.997 and 17.70, as well as showing relatively low root mean square error values at 0.44 N mm^−2^ during global modeling, demonstrating excellent predictive performance.

**Conclusions:**

This study delivered a successful attempt for rapid and precise prediction of rind penetrometer resistance and breaking force in sugarcane stalk by NIRS assay. These established models can be used to improve phenotyping jobs for sugarcane germplasm on a large scale.

**Supplementary Information:**

The online version contains supplementary material available at 10.1186/s13007-023-01076-0.

## Background

Sugarcane (*Saccharum spp.*) is a perennial C4 crop well adapted to subtropical and tropical regions for sugar and bioethanol production [1–3]. Since sugar and biofuel demand has increased, emphasis has been put on maximizing per-area production and standardizing agronomic practices to achieve optimal yields [4, 5]. Stalk lodging (breakage or bend of stalks before harvest) is one of the main factors that largely restricts sugarcane production, estimating 10% to 20% yield lost annually [6, 7]. In the past few years, studies have explored the lodging resistance of plants from the view of field management practices, plant architecture [8], and plant biomechanics [9]. Efforts also have been made to improve lodging resistance through genetic improvement [10, 11], but the results have been limited due to the complex and multifactorial nature of lodging traits and the low efficiency of accurate characterization.

Generally, the stem lodging resistance of cereal crops can be evaluated by mechanical strength according to measuring two types of indicators (rind penetrometer resistance and breaking force) values [12–15]. Breaking force is normally applied in small cereal crops with hollow stems such as wheat and rice [16–18], while rind penetrometer resistance (RPR) seems more suitable for large cereal crops such as corn and sorghum [19, 20]. In contrast to the crops described above, sugarcane is a stalk harvest crop in which little carbohydrate is redistributed in the stem during maturation [21, 22]. Sugarcane stalk lodging resistance is closely related to its biological and physical properties, such as stalk height, diameter, mechanical strength, hardness of barks, and fiber content [23, 24]. Sugarcane stem failure can be divided into greensnap and stalk lodging [25]. Greensnap refers to stalk breakage at the young stem internode in the face of external force, whereas stalk lodging refers to stem internode buckling at the mature stem internode when the stalk could not support its weight or face external force [26]. Typically, greensnap occurs less frequently than lodging, but once it occurs, it can cause significant damage to sugarcane, particularly during severe weather conditions, such as high-intensity typhoons [25, 27]. From a plant breeding or phenotyping perspective, both types of stem failure should be distinct. As such, in our recent study, breaking force and rind penetrometer resistance (RPR) were successfully applied for characterizing these two types of stem failure [28]. However, laboratory-based mechanical phenotyping jobs require a considerable amount of time, making it difficult to apply for high-throughput phenotyping jobs.

Near-infrared spectroscopy (NIRS) is a fast, simple, and high-efficient analytical technique that integrates measurement, data collection, and analysis altogether to predict the properties of samples [29]. By chemometric calibration, a regression model is established between the spectrum and the measured value, enabling qualitative and quantitative analysis to be carried out [30–33]. In recent years, NIRS has been widely used in the agriculture, food, petrochemical, and pharmaceutical fields [34]. Such as high-throughput screening of plant biomass samples [35], quick and large-scale screen the target traits of crops [36], and analysis of multiple traits in crop breeding [37–39]. In our previous study, NIRS has been successfully applied for sugarcane stalk quality assessment in terms of moisture, soluble sugar, insoluble residue, and the corresponding fundamental ratios [40]. Besides, cell wall features calibration models have also been developed for the relevant genetic development [41]. There appears to be the possibility of screening large-scale sugarcane germplasm for sugarcane breeding using these proposed methods. However, no study has so far attempted to determine stalk mechanical properties with NIRS in sugarcane or other crops.

The objective of this study was to develop a rapid NIRS assay for sugarcane rind penetrometer resistance (RPR) and breaking force determination. A large number of sugarcane genotypes were collected from two consecutive years (2019 and 2020). Using well-established laboratory methods for determining RPR and breaking force, a NIRS assay was developed for predicting these two types of mechanical strength in sugarcane stalks through the standard calibration process. Thus, this study provided a high-throughput NIRS assay for mechanical strength characterization in sugarcane, which could be integrated as a system project with our previous studies for multipurpose precision breeding.

## Results

### Precise laboratory assay for mechanical strength determination in sugarcane stalks

**Fig.1:**
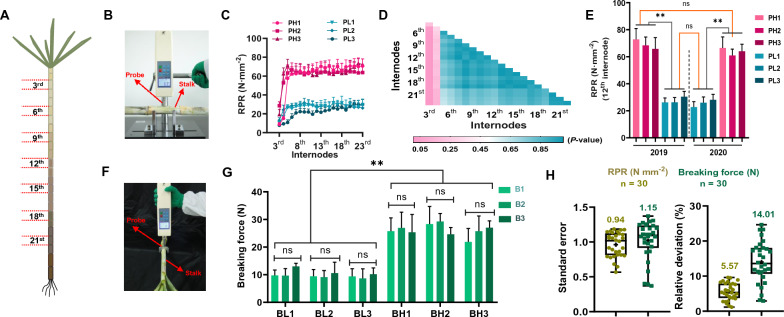
High-performance laboratory analytical methods for measuring mechanical strengths in sugarcane stalk. **A** Schematic diagram of multi-internodes of sugarcane. **B** RPR measurement. **C** Distribution of RPR value in different internodes of sugarcane genotypes. **D** Multiple comparison analysis of internodes RPR in sugarcane (values were calculated by one-way ANOVA and LSD test, n = 6, α ≤ 0.05). RPR: rind penetrometer resistance. **E** Comparison analysis of RPR values in sugarcane genotypes across different years. PH1-PH3: genotypes with high RPR; PL1-PL3: genotypes with low RPR. **F** Breaking force measurement. **G** Comparison analysis of breaking force in sugarcane genotypes. BH1-BH3: genotypes with high breaking force; BL1-BL3: genotypes with low breaking force; B1-B3: sugarcane planted at three different fields. ** indicated statistically significant differences at *p* < 0.01 level. **H** Standard error of RPR and breaking force measurement

In this study, we used an Instron Universal Testing Machine to determine the rind penetrometer resistance (RPR) of the sugarcane stalk in the laboratory (Fig. 1B). As sugarcane stalks have multiple internodes (Fig. 1A), we compared the RPR of sugarcane stalks from the different internodes of selected genotypes that had contrasted higher and lower RPR. It was observed that RPR increased dramatically from the 3rd internode to the 5th internode (Fig. 1C). It is noteworthy, that no significant difference was observed from the 7th internode to the 23rd internode (Fig. 1C). Additionally, the internode RPR showed similar variation patterns among different genotypes (Fig. 1C). Therefore, we calculated the differences in RPR among genotypes within the same internode. From the 5th to the 23rd internode, stable differences were observed between genotypes with high and low RPR (Additional file 1: Annex 1). Furthermore, the results of the multiple comparison analysis of RPR between different internodes revealed that none of them showed significant differences, except the 3rd and 4th internode (Fig. 1D). It was suggested that any internode except the third and fourth one can be used as a representative internode for measuring RPR. As a means of verification, we measured the RPR of representative genotypes at the 12th internode in 2019 and 2020, respectively. Notably, significant and stable differences were detected between high and low RPR genotypes and no detectable difference was observed within high or low RPR genotypes between the two years (Fig. 1E).

Based on phenotypic observations in the field, we observed that greensnap occurred only in the younger node (the 3rd node) (Additional file 1: Annex 2). To examine this phenomenon in more detail, we determined the breaking force across a large number of sugarcane genotypes (Fig. 1F). Several sugarcane genotypes with high and low breaking forces were selected to determine their breaking force in different environments. Accordingly, there was no significant difference in the breaking force of a given genotype in different environments, but there was a significant difference between those genotypes with a higher and a lower breaking force (Fig. 1G). Considering these results, it was concluded that breaking force is under strong genetic control, hence selection against this trait is possible.

Additionally, we calculated the standard errors for both types of mechanical strengths under laboratory conditions separately. It was determined that RPR displayed a smaller measurement error compared to break force. The observed standard error ranged from 0.57 to 1.19 N mm^−2^ for RPR and 0.37 to 1.37 N for the breaking force, with the mean value at 0.94 and 1.15 respectively (Fig. 1H). The relative deviation was calculated at the mean value of 5.57% and 14.01% for RPR and breaking force, respectively (Fig. 1H). The results validated the reliability of these approaches, confirming that the method could be effectively used to analyze both RPR and breaking force in sugarcane stalks accurately and suitably. In particular, the RPR exhibited a greater level of measurement accuracy and stability, which greatly contributed to the development of NIRS models.

### Mechanical strength determination in collected sugarcane genotypes

**Fig. 2:**
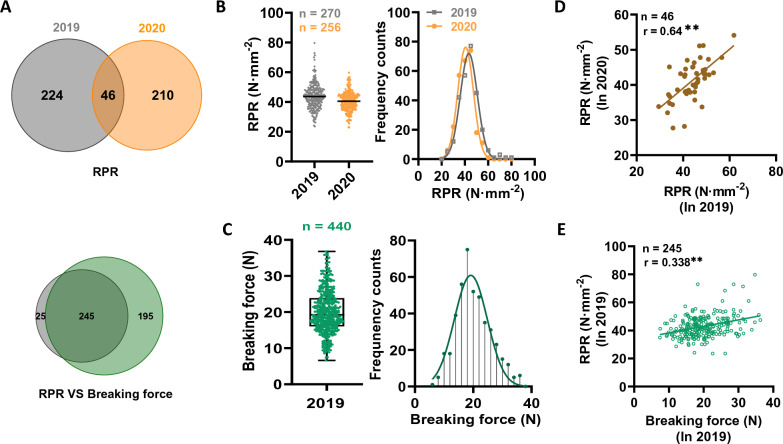
Variation of stalk mechanical strength in sugarcane population. **A** Venn diagram representing the number of sugarcane genotypes used for mechanical strength measurement. **B**, **C** Variated distribution of RPR (**B**) and breaking force (**C**) in sugarcane stalks. **D** Correlation analysis of sugarcane stalk RPR in 2019 and 2020. **E** Correlation analysis between RPR and breaking force in sugarcane genotypes. *RPR* rind penetrometer resistance. ** indicated statistically significant correlation at *p* < 0.01 level

By using the established laboratory assay described above, we evaluated the RPR and breaking forces of sugarcane germplasm in order to obtain the genotype observations (true values) for the NIRS modeling. Specifically, RPR data were collected on 270 sugarcane genotypes in 2019 and 256 genotypes in 2020, with 46 genotypes being shared between both years (Fig. 2A). Breaking force measurements were conducted on 440 sugarcane genotypes, of which 245 genotypes were also measured with RPR (Fig. 2A). Consequently, we observed wide variations in RPR among sugarcane genotypes that followed a normal distribution in both years (Fig. 2B). A detailed analysis of this data reveals that it ranged from 23.5 to 79.7 N mm^−2^ in 2019, but decreased in 2020, ranging from 22.8 to 59.7 N mm^−2^ (Additional file 1: Annex 3). Similarly, large variations were observed for breaking force in sugarcane genotypes as well, which presented a normal distribution ranging between 6.6 N and 32.8 N (Fig. 2C; Additional file 1: Annex 3).

In addition, a correlation analysis was conducted between the RPR values measured in 2019 and 2020 based on these 46 shared genotypes. A significant positive correlation result was observed at *p* < 0.01 level (Fig. 2D). Using these 245 genotypes that were shared for both RPR and breaking force determination, we performed a correlation analysis between these two types of mechanical strength. Interestingly, these two types of force traits (RPR and breaking force) had a significant correlation coefficient of 0.338 at *p* < 0.01 level, indicating that they are probably associated (Fig. 2E).

### NIRS data characterization in collected sugarcane stalks

**Fig. 3:**
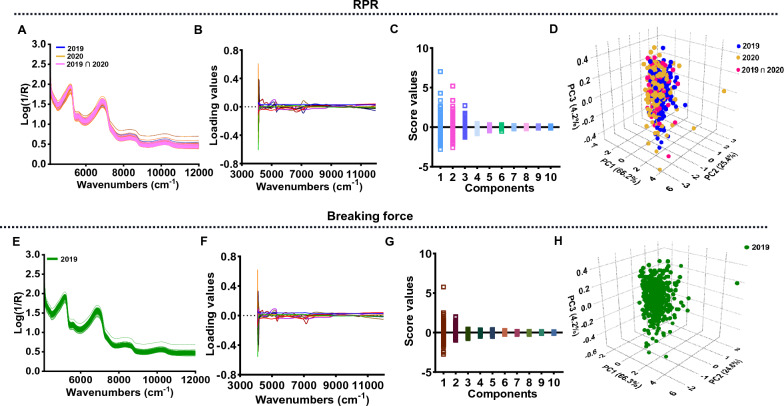
Near-infrared spectral characterizations in sugarcane genotypes. **A**, **E** Original spectral of the samples used for RPR (**A**) and breaking force (**E**). RPR: rind penetrometer resistance. **B**, **F** The first 10 principal components for near-infrared spectral characterization. **C**, **G** Genotype variation in each principal component in the sugarcane. **D**, **H** 3D view of the collected sugarcane genotypes via PCA

Near-infrared spectroscopic collection was conducted immediately after mechanical strength evaluation in the laboratory for each of these sugarcane genotypes. According to Fig. 3A and E, the near-infrared reflectance values of all sugarcane genotypes fluctuated within the normal range. Principal components analysis (PCA) was applied for NIRS data characterization. During PCA, the top ten principal components that could explain 99.6% and 99.7% of the variation were selected to characterize the genotype distribution for RPR and breaking force, respectively (Fig. 3B and F). Each variable in the spectra used for RPR and breaking force modeling can be found to show different variations in the selected principal components (PCs), especially for the first five PCs (Fig. 3C and G). Afterwards, these first three PCs were applied for a 3D observation of the genotype distribution. Even though genotypes were collected from different years (2019 and 2020) for RPR determination, no significant discrimination was observed between the spectra (Fig. 3D). Observations of the spectra of these shared 46 genotypes revealed a smaller global distance (GH), suggesting a high level of similarity between them (Fig. 3D). In the case of these genotypes used for breaking force measurements, the first three principal components accounted for 95.1% of the total and displayed a continuous distribution (Fig. 3H), suggesting that these genotypes can be incorporated into a global calibration population for NIRS.

### Determination of calibration and external validation sets

**Fig. 4:**
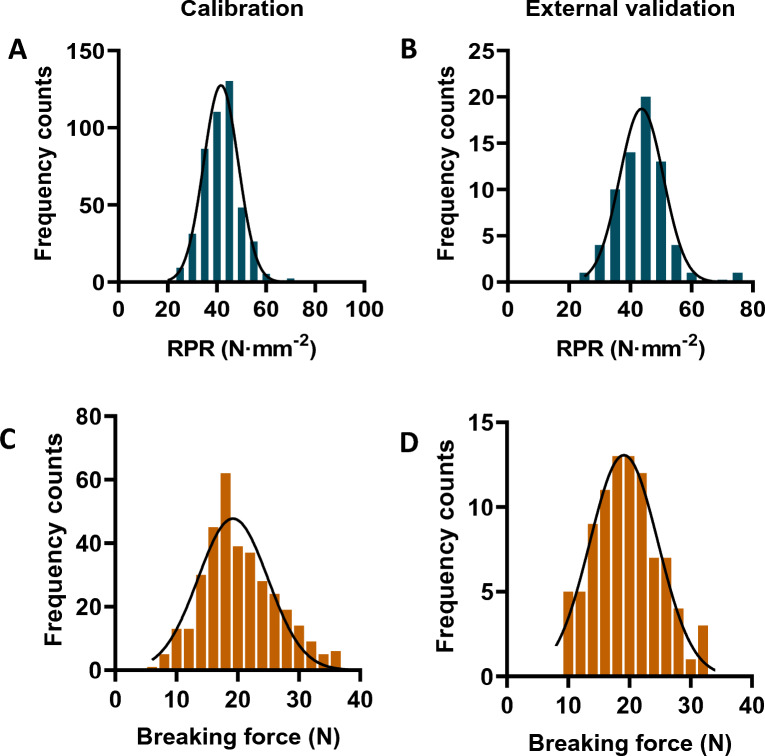
Statistics of sugarcane genotypes for calibration and external validation. **A**, **C** Frequency distribution of genotypes for calibration of RPR (**A**) and breaking force (**C**). **B**, **D** Frequency distribution of genotypes for external validation of RPR (**B**) and breaking force (**D**)

For NIRS modeling, these sugarcane genotypes were divided into two sets, one was used for calibration and the other was used for external validation. In the case of NIRS modeling of RPR, a total of 458 genotypes were randomly selected into the calibration set, and the remaining 68 genotypes formed the external validation sets (Fig. 4; Additional file 1: Annex 4). In terms of breaking force, 90 genotypes were used for external validation and 350 genotypes for calibration (Fig. 4; Additional file 1: Annex 4). An analysis of descriptive statistics was conducted to compare the calibration and external validation sets. It is noteworthy, that the minimum and maximum values at both ends of the external validation set were included in the calibration set to ensure that the model is both accurate and practical (Additional file 1: Annex 4). Additionally, RPR and breaking force displayed normal distributions for both calibration and external validation sets (Fig. 4). All statistical distributions across calibration and external validation were comparable, suggesting that it is feasible to obtain accurate predictive equations.

### Stalk mechanical strength modeling and evaluation

We applied NIRS modeling independently to two different types of mechanical strength indicators (RPR and breaking force) of sugarcane stalks. According to RPR calibration, we observed that the* R*^*2*^ was reaching 0.997, the RPD value was reaching 19.60, as well as a relatively low RMSEC value at 0.40 N (Table 1). In terms of NIRS calibration for breaking force, although the modeling parameters were not as good as for RPR, they still demonstrated excellent fitting with *R*^*2*^, RPD, and RMSEC values of 0.880, 2.88, and 2.15 N, respectively (Table 1). Further, internal cross-validation was conducted to assess these obtained models. During internal cross-validation, the genotypes were divided into various groups, some of which were chosen at random from the calibration sets for cross-validation, which provides the root mean square error of cross-validation (RMSECV) and coefficient determination (*R*^*2*^*cv*), respectively, for model evaluation. According to our results, a high *R*^*2*^*cv* (0.991), RPD (10.30) value, and a relatively low RMSECV (0.74 N) were observed for the model of RPR prediction. Likewise, the *R*^*2*^*cv* value was 0.830, RPD was 2.42, and RMSECV was 2.51 N for the models of RPR prediction (Table 1). In this case, the RPR model showed better predictive performance than the breaking force model, which was consistent with the calibration results.

**Table 1:** Calibration and external validation statistics for RPR and breaking force in sugarcane stalks

	Calibration									Cross-validation			External validation			
	Rank	N	SCM	Spectrum range	Mean	SD	RMSEC	*R*^*2*^	RPD	RMSECV	*R*^*2*^_*cv*_	RPD	N	RMSEP	*R*^*2*^_*ev*_	RPD
RPR (N·mm^−2^)	23	458	SNV	5669.7–9626.9, 10406–11200.6	42.0	7.62	0.40	0.997	19.60	0.74	0.991	10.30	68	0.76	0.990	10.20
Breaking force (N)	12	350	FD + SNV	4882.9–6464.2, 8037.9–9626.9	20.3	6.08	2.15	0.880	2.88	2.51	0.830	2.42	90	2.08	0.849	2.58

*RPR*, rind penetrometer resistance; *Rank*, latent variables used in PLS regression; *N*, genotype number; SCM, scatter correction methods; SD, standard deviation of reference value; RMSEC, root mean square error of calibration;* R*^*2*^, determination coefficient of calibration; *R*^*2*^_cv_, determination coefficient of cross-validation; RMSECV, root mean square error of cross-validation; RMSEP, root mean square error of external validation;* R*^*2*^_ev_, determination coefficient of external validation; RPD, the ratio of prediction to deviation; SNV, standard normal variate, *FD*, first derivative, *FD + SNV*, a combination of FD and SNV

Additionally, the models were subjected to external validation as an independent test to assess their performance. Similarly, for model evaluation, root mean square error of external validation (RMSEP), coefficient determination (*R*^*2*^*ev*), and the ratio of prediction to deviation (RPD) were calculated. It was found that, in this context, all models for RPR and breaking force showed *R*^*2*^*ev* values of above 0.849 and RPD values well above 2.5 (Table 1). A notable feature of the model for RPR was that the coefficient of determination and ratio of prediction to deviation remained constant at 0.990 and 10.20, respectively (Table 1), in accordance with the good performance observed during calibration and internal cross-validation, suggesting their high prediction performance.

### Global modeling of the stalk mechanical strength

**Fig.5:**
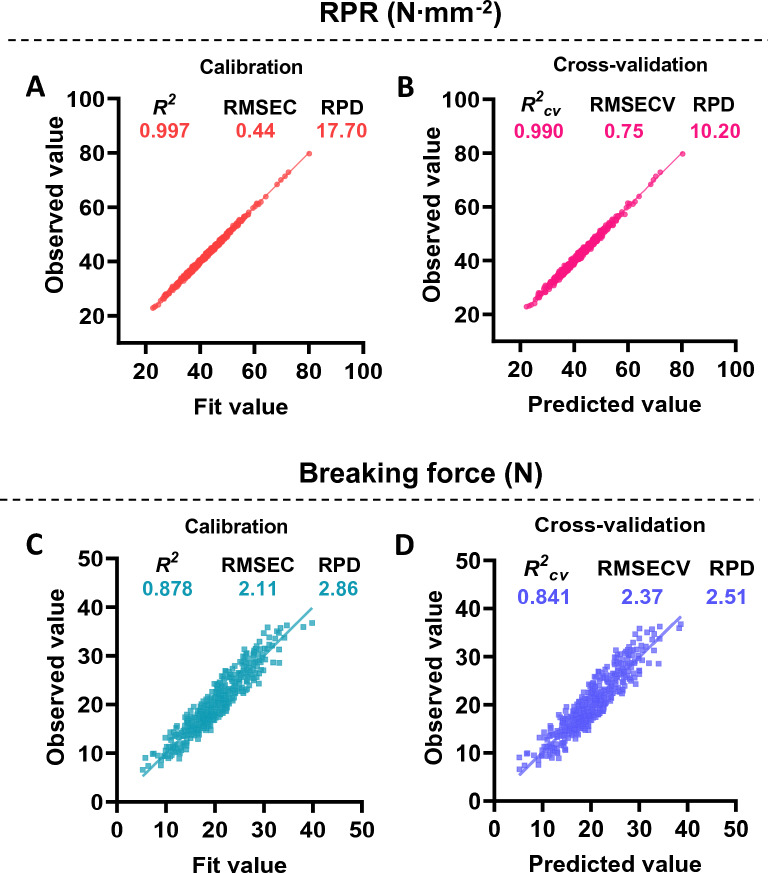
Correlation between the fit (predicted) value and observed value for stalk mechanical strengths in sugarcane. **A**, **C** Calibration for RPR (**A**) and breaking force (**C**); **B**, **D** Cross-validation for RPR (**B**) and breaking force (**D**). RPR: rind penetrometer resistance; *R*^2^, determination coefficient of calibration; *R*^*2*^*cv*, determination coefficient of cross-validation; RMSEC, root mean square error of calibration; RMSECV, root mean square error of cross-validation; RPD, ratio of prediction to deviation

We then combined the external validation set with the calibration set to form an integrated calibration set to perform an integrative calibration analysis to gain higher performance model predictions. The results showed that the parameters of the new RPR model did not significantly improve, but the prediction performance remained extremely high (Fig. 5A and B; Additional file 1: Annex 5). A slightly improved *R*^*2*^*cv* (0.841) and RPD (2.51) values were found for the breaking force model (Table 1; Fig. 5C and D). Despite the high correlation between the true value and the fit (predicted) value (Fig. 5C and D), it is evident that the obtained breaking force model can provide reliable predictions.

## Discussion

Lodging is one of the major problems that affect growth and potential yield in agricultural crops [16]. In particular, sugarcane is a large crop that is highly susceptible to stalk lodging, which results in approximately ten percent to twenty percent of sugarcane yield being lost annually [42]. However, lodging is a very complex trait that is affected by a number of factors. In regard to stem lodging, it is related to the mechanical properties of the stem, as well as to its biological characteristics, such as height, stem diameter, weight, etc. [43]. Generally, crop lodging resistance can be improved by either reducing plant height or increasing stalk mechanical strength [44]. For instance, by breeding dwarf varieties, the first green revolution greatly reduced main grain crop failure [45]. However, due to the stalk-harvesting nature of sugarcane, this strategy was not feasible. The efficient way to increase its resistance to stalk lodging is to enhance its mechanical strength [46].

In general, the RPR and breaking force of the stalk are reliable indicators of the mechanical strength of the stalk [47]. In crop breeding, RPR and breaking force have been used to indirectly screen and develop lodging-resistant varieties [48–50]. Unfortunately, due to the lack of an efficient method for accurately characterizing RPR or breaking force, lodging-resistant breeding in sugarcane has largely been limited.

This study was designed to develop a method for the rapid and precise prediction of RPR and breaking force in sugarcane stalks via NIRS modeling. Firstly, a precise laboratory analytical method was successfully established for determining the RPR and breaking force in sugarcane (Fig. 1). Accordingly, substantial variations in RPR and breaking force were observed in collected sugarcane germplasms (Fig. 2B and C), which was the crucial element for accurate NIRS modeling in this study. Besides, highly significant correlations were observed between breaking force and RPR (Fig. 2E), indicating there would be a certain internal relationship between these two types of mechanical strength. It has been observed that RPR is inferior in determining mechanical strength in breeding populations, which makes it difficult to screen for lodging susceptible genotypes [51, 52]. Hence, in multi-internode crops, RPR along with breaking force are used as lodging determination index [53, 54]. These indices have been extensively used to measure stalk lodging in maize [55–57]. However, little study has been available to evaluate sugarcane genotypes based on these lodging indices. It is evident from the current study that the sugarcane genotype having a strong breaking force in the young node also exhibited higher RPR in mature internodes. In view of this closely linked relationship, sugarcane breeding programs aimed at increasing mechanical strength through collaborative improvements in breaking force and RPR were supported.

Due to a wide range of genetic variation in collected sugarcane genotypes, a continuous distribution of NIR spectra was obtained (Fig. 3), which provides a well-founded basis for NIRS modeling. As we expected, the high performance of NIRS models for RPR and breaking force determination were obtained based on a PLS calibration analysis (Table 1). Particularly, the parameters of the prediction model for RPR were much higher than those of the prediction model for breaking force (Table 1). A possible explanation for the relatively low *R*^*2*^ and RPD values observed in the breaking force prediction model could be due to the relatively large standard error observed in its laboratory determination method (Fig. 1H). Besides, only one year's worth of data was used for the modeling process (Table 1; Fig. 2), which further limited the ability to calibrate the breaking force. In spite of this, the prediction model for breaking force remains relevant, and the prediction performance can be further improved by adding more samples with a variety of features.

Generally, models having RPD > 2.5 were classified as “Fair”, which were considered effective for screening applications [58]. In this study, all the obtained models displayed RPD values over 2.5, along with highly correlated true and predicted values in the calibration, internal cross-validation, and external validation (Table 1; Fig. 5), suggesting their sufficient prediction capability [36, 59, 60]. Particularly, the model for RPR characterization displayed the RPD values as high as 19.60 during the calibration process, with an *R*^*2*^ value of 0.997, indicating excellent application performance (Table 1; Fig. 5). In addition, we also attempted to establish the RPR model using genotypes collected in 2019 and validate it using genotypes collected in 2020. In spite of high *R*^*2*^ and *R*^*2*^*cv* values observed during calibration and cross-validation, a relatively low *R*^*2*^*ev* value of 0.894 was observed during external validation, which further indicates that accuracy and stability of NIRS models are limited when one year's data is utilized for modeling (Additional file 1: Fig. S3). It was unexpected that the performance of the RPR prediction model failed to improve when genotypes were added from an external validation set. This may be due to the inclusion of some outlier genotypes in the external validation set. Despite this, the final calibration results showed that all the models performed exceptionally well in their respective applications. By comparing the results of all models generated in this study, the newly integrated calibration model appears to offer good potential for high-throughput screening of excellent germplasm from large-scale sugarcane genotypes.

According to multiple linear regression analysis, loading reflects the amount of contribution each factor makes to the dependent variable. Based on our general modeling and global modeling analyses, we found a consistent selection of wavelength ranges and spectrum pretreatment methods for both types of mechanical strength (Table 1 and Additional file 1: Table S3). In spite of different calibration strategies, similar loading results were observed for both types of mechanical strength (Additional file 1: Fig. S4). Regarding RPR, consensus high loading values were observed at the wavelength of 6302, 6665, 6626, 7227, 9534, and 10406 cm^−1^ (Additional file 1: Fig. S4A and C). Whereas, for the breaking force calibration, the conserved high loading values were observed at the wavelength of 4906, 4999, 5785, 8709, and 9349 cm^−1^ (Additional file 1: Fig. S4B and D). These selected wavelengths of the near-infrared spectrum should play key roles in mechanical strength modeling in sugarcane.

For the purpose of breeding sugarcane for lodging resistance, we emphasize the importance of assessing sugarcane stalk mechanical strength; however, we are also aware that there are many factors contributing to lodging, and stalk mechanical strength cannot be used as the only indicator for sugarcane lodging traits characterization. As the scope of this study was limited to establishing the methods for the evaluation of RPR and breaking force in sugarcane stalks, other parameters will require further investigation, such as crushing strength and bending strength. Additionally, the RPR assay developed in this study could be used to characterize stalk tissue hardness. It is well known that there is a complex carbon source allocation process in the stalk tissue during the development of sugarcane. Source–sink relationships during crop development have been emphasized to influence the sugar accumulation in sugarcane [61–63]. It is likely that stalk tissue will have a higher hardness if more carbon sources are allocated for the synthesis of the cell wall, but sugar accumulation may be negatively affected. In this manner, RPR may be considered as one of the indicators for detecting carbon allocation modes in sugarcane. Those genotypes with higher RPR represent more carbon source allocation to the cell wall. Alternatively, a low RPR indicates that more carbon sources are allocated to parenchyma cells for the purpose of accumulating sugars. Interestingly, over-expression of an essential gene (*OsSUS3*) related to carbon partition leads to largely enhanced lodging resistance by distinctively altering lignocellulose features in rice, but little affects yield traits [64]. A recent study has explored the feasibility of large-scale screening methods for both carbohydrate features and lodging resistance prediction by means of near-infrared spectroscopic techniques [65]. Consequently, the RPR determination method developed here has a promising application in the study of carbon source partitioning within sugarcane.

## Conclusions

This study developed a high-throughput analysis method based on NIRS to estimate sugarcane stalk mechanical strength for the first time. We conducted a large-scale study on the precise evaluation of the mechanical strength of sugarcane germplasm resources based on the establishment of laboratory methods for the determination of sugarcane RPR and breaking force. By combining mechanical strength data with NIRS data derived from these sugarcane germplasm resources, calibration models for predicting RPR and breaking force were developed via chemometrics analysis. Most of the models exhibited perfect prediction abilities with high values of *R*^*2*^, *R*^*2*^_cv_, *R*^*2*^_ev,_ and RPD, particularly the equation for RPR characterization displayed an *R*^*2*^ value as high as 0.997, suggesting excellent application performance. The findings of this study provided reliable technical support and solutions for high-throughput screening of sugarcane breeding and other research areas.

## Materials and methods

### Sample processing

Sugarcane germplasms were collected and planted in the Fusui experimental field of Guangxi University, China (107°47′17.66′′ E, 22°31′5.85′′ N). Sugarcane stalks were harvested at the mature stage (270–280 d after planting or ratooning) in the years 2019 and 2020. In detail, 270 and 256 genotypes were collected in 2019 and 2020, respectively, for RPR determination. A total of 440 sugarcane genotypes were used for breaking force determination in 2019. Six stalks were randomly selected from each genotype for mechanical strength determination and further NIRS analysis after removing leaves but keeping young tips.

### Mechanical strength determination in sugarcane stalks

RPR determination: The Instron Universal Testing Machine (YYD-1) equipped with a circular puncture probe (Sect. 1 mm^2^) was used to determine the RPR of sugarcane stalks. In detail, five independent positions in the middle of the 12th and 15th internode were detected by puncture probe in 2019 and 2020, respectively, and the peak value of each position was recorded. The average RPR value of each internode was calculated on these collected data after eliminating the maximum and minimum values. Six biological replications were performed for each genotype.

Breaking force determination: The Instron Universal Testing Machine (YYD-1) configured with an arc probe was applied for breaking force measurement. Briefly, the sugarcane with a young tip was fixed flatting on the loading platform, where the 3rd internode was extended out for breaking force detection. The arc probe was kept perpendicular to push the fourth internode until it broke, and the peak force value was recorded. Six biological replications were performed for each genotype.

### Online near-infrared spectral data source

After mechanical properties measurement, the collected six stalks of each genotype were used for near-infrared spectral detecting. Sample pretreatment and data collection followed a standard pipeline as described in our previous studies [40]. In brief, sugarcane stalks were shredded using DM540 (IRBI Machines & Equipment Ltd, Brazil), and the shredded fresh sample was immediately transported to the CPS system (Cane presentation system, Bruker Optik GmbH, Germany) by a conveyor belt, where the near-infrared spectral data of each genotype was online collected by MATRIX-F (Bruker Optik GmbH, Germany) system. During the shredding process, none of the sugarcane stalk components were lost, and the moisture in the shredded bagasse was retained. The obtained continuous near-infrared spectral reflectance values were then averaged for further analysis.

### NIRS data processing and calibration

The OPUS spectroscopy software (version 7.8, Bruker Optik GmbH, Germany) was used for data processing and NIRS calibration. Before NIRS modeling, the collected sugarcane germplasms were randomly divided into calibration and validation sets in a 4:1 ratio. Among them, the calibration set was used for modeling, and the validation set was applied for external validation. A principal component analysis (PCA) was performed to identify the spectral outliers, as well as determine the structure and variability of the spectral population. As described by Wang et al. [40], pretreatment and the wavelength range selection of the raw spectral data were performed before calibration to solve the problems associated with the overlapping peaks and baseline correction. Briefly, several spectral pretreatment methods were used in OPUS software, namely constant offset elimination (COE), straight-line subtraction (SSL), standard normal variate (SNV), Min–Max normalization (MMN), multiplicative scattering correction (MSC), first derivative (FD), second derivative (SED), a combination of the first derivative and straight-line subtraction (FD + SSL), a combination of the first derivative and standard normal variate (FD + SNV), and a combination of the first derivative and multiplicative scattering correction (FD + MSC). A default setting in OPUS software was used to select the wavelength range. A combination in terms of wavelength range selection and spectrum pretreatment was made to obtain calibration models in PLS analysis. The final matrix with the dimension of 618 × 526 and 413 × 440 were applied for RPR and breaking force model calibration, respectively. Internal cross-validation and external validation were carried out to test the performance of the generated models. During internal cross-validation, the calibration set was divided into several groups according to the default parameters available in the OPUS software. Each group was then validated using a calibration developed on other genotypes. Finally, validation errors were combined into a standard error of cross-validation for model performance evaluation. The best model was selected according to the high coefficient of determination of the calibration/internal cross-validation/external validation (*R*^*2*^/*R*^*2*^_cv_/*R*^*2*^_ev_), low root mean square error of calibration/internal cross-validation/external validation (RMSEC/RMSECV/RMSEP), and high ratio of prediction to deviation (RPD) values.

### Supplementary Information

**Additional file 1: Fig. S1**. Analysis of RPR in sugarcane internodes, differences in RPR between the same internodes of different genotypes (values represent the mean ± SD, n=3, **p＜0.01). **Fig. S2**. Greensnap of sugarcane in the field. **Table. S1**. Diversity of mechanical strength in the sugarcane germplasms. **Fig. S3**. Correlation between the fit (predicted) value and observed value for RPR in sugarcane. A-B: Calibration (A) and internal cross-validation (B) for RPR by using 270 genotypes collected in 2019. (C) External validation for RPR by using 256 genotypes collected in 2020. *R*^2^, determination coefficient of calibration; *R*^*2*^*cv*, determination coefficient of cross-validation; *R*^*2*^_*ev*_, determination coefficient of external validation; RMSEC, root mean square error of calibration; RMSECV, root mean square error of cross-validation; RMSEP, root mean square error of external validation; RPD, the ratio of prediction to deviation. **Fig. S4**. Regression coefficient (loadings) of the optimal NIRS model. A-B: distribution of loading values for RPR (A) and breaking force (B) upon general modeling; C-D: distribution of loading values for RPR (C) and breaking force (D) upon global modeling. **Table. S2**. Calibration and external validation statistics for RPR and breaking force in sugarcane. **Table. S3**. Statistics for generated equations for RPR and breaking force in sugarcane stalks.

## Data Availability

All data generated or analyzed during this study are included in this published article [and its supplementary information files].
