# Supplementary material for: A high-throughput phenotyping method for sugarcane rind penetrometer resistance and breaking force characterization by near-infrared spectroscopy
Source: Plant Methods. 2023 Sep 28;19:101. doi: 10.1186/s13007-023-01076-0 (PMC10540387; doi:10.1186/s13007-023-01076-0)
Supplement: Supplementary file 1 — Additional file 1: Fig. S1. Analysis of RPR in sugarcane internodes, differences in RPR between the same internodes of different genotypes (values represent the mean ± SD, n=3, **p＜0.01). Fig. S2. Greensnap of sugarcane in the field. Table. S1. Diversity of mechanical strength in the sugarcane germplasms. Fig. S3. Correlation between the fit (predicted) value and observed value for RPR in sugarcane. A-B: Calibration (A) and internal cross-validation (B) for RPR by using 270 genotypes collected in 2019. (C) External validation for RPR by using 256 genotypes collected in 2020. R2, determination coefficient of calibration; R2cv, determination coefficient of cross-validation; R2ev, determination coefficient of external validation; RMSEC, root mean square error of calibration; RMSECV, root mean square error of cross-validation; RMSEP, root mean square error of external validation; RPD, the ratio of prediction to deviation. Fig. S4. Regression coefficient (loadings) of the optimal NIRS model. A-B: distribution of loading values for RPR (A) and breaking force (B) upon general modeling; C-D: distribution of loading values for RPR (C) and breaking force (D) upon global modeling. Table. S2. Calibration and external validation statistics for RPR and breaking force in sugarcane. Table. S3. Statistics for generated equations for RPR and breaking force in sugarcane stalks. [file 13007_2023_1076_MOESM1_ESM.pptx]

## Slide 1
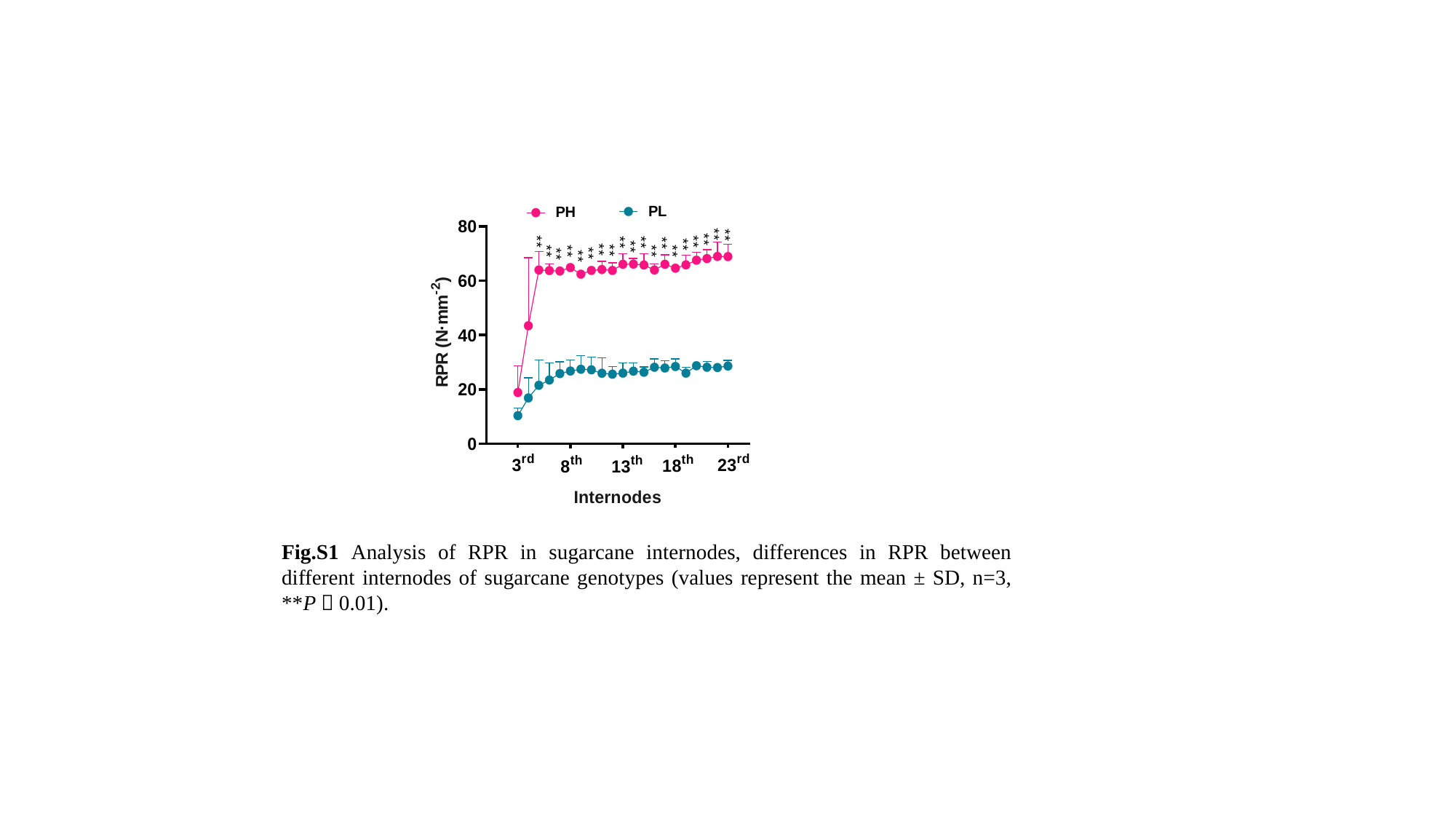

Fig.S1 Analysis of RPR in sugarcane internodes, differences in RPR between different internodes of sugarcane genotypes (values represent the mean ± SD, n=3, **P＜0.01).

## Slide 2
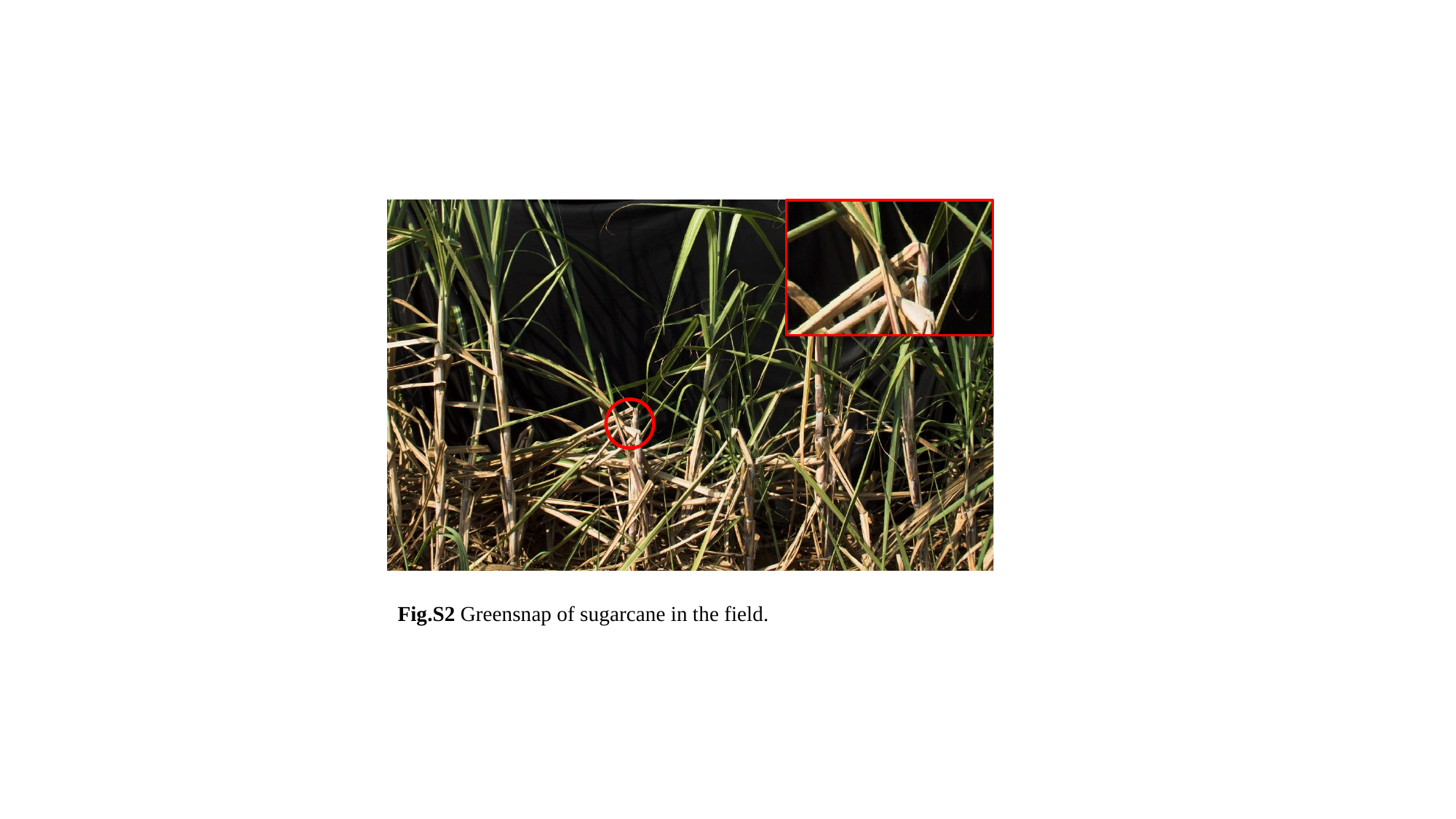

Fig.S2 Greensnap of sugarcane in the field.

## Slide 3
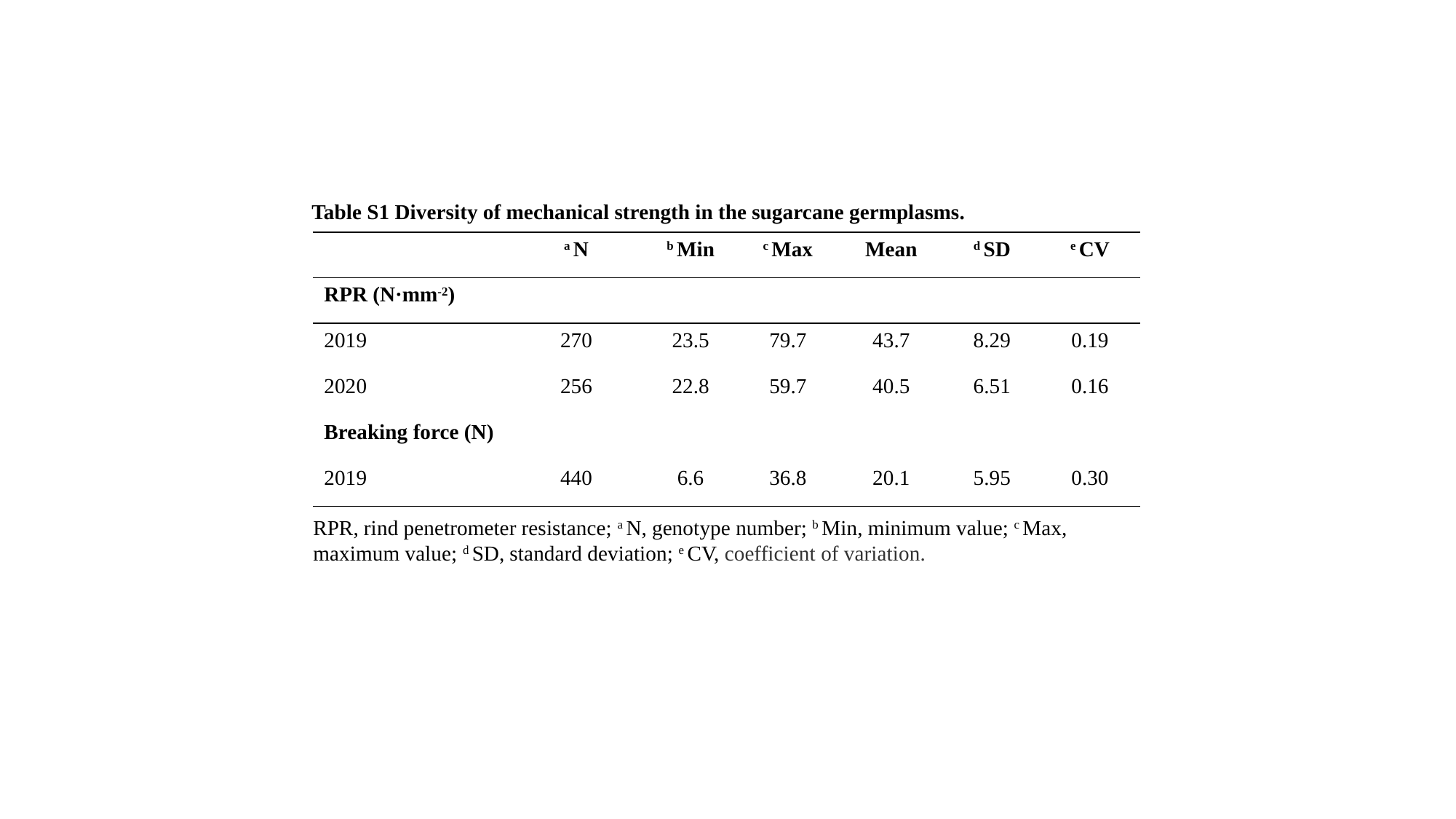

Table S1 Diversity of mechanical strength in the sugarcane germplasms.
| | a N | b Min | c Max | Mean | d SD | e CV |
| --- | --- | --- | --- | --- | --- | --- |
| RPR (N·mm-2) | | | | | | |
| 2019 | 270 | 23.5 | 79.7 | 43.7 | 8.29 | 0.19 |
| 2020 | 256 | 22.8 | 59.7 | 40.5 | 6.51 | 0.16 |
| Breaking force (N) | | | | | | |
| 2019 | 440 | 6.6 | 36.8 | 20.1 | 5.95 | 0.30 |
RPR, rind penetrometer resistance; a N, genotype number; b Min, minimum value; c Max, maximum value; d SD, standard deviation; e CV, coefficient of variation.

## Slide 4
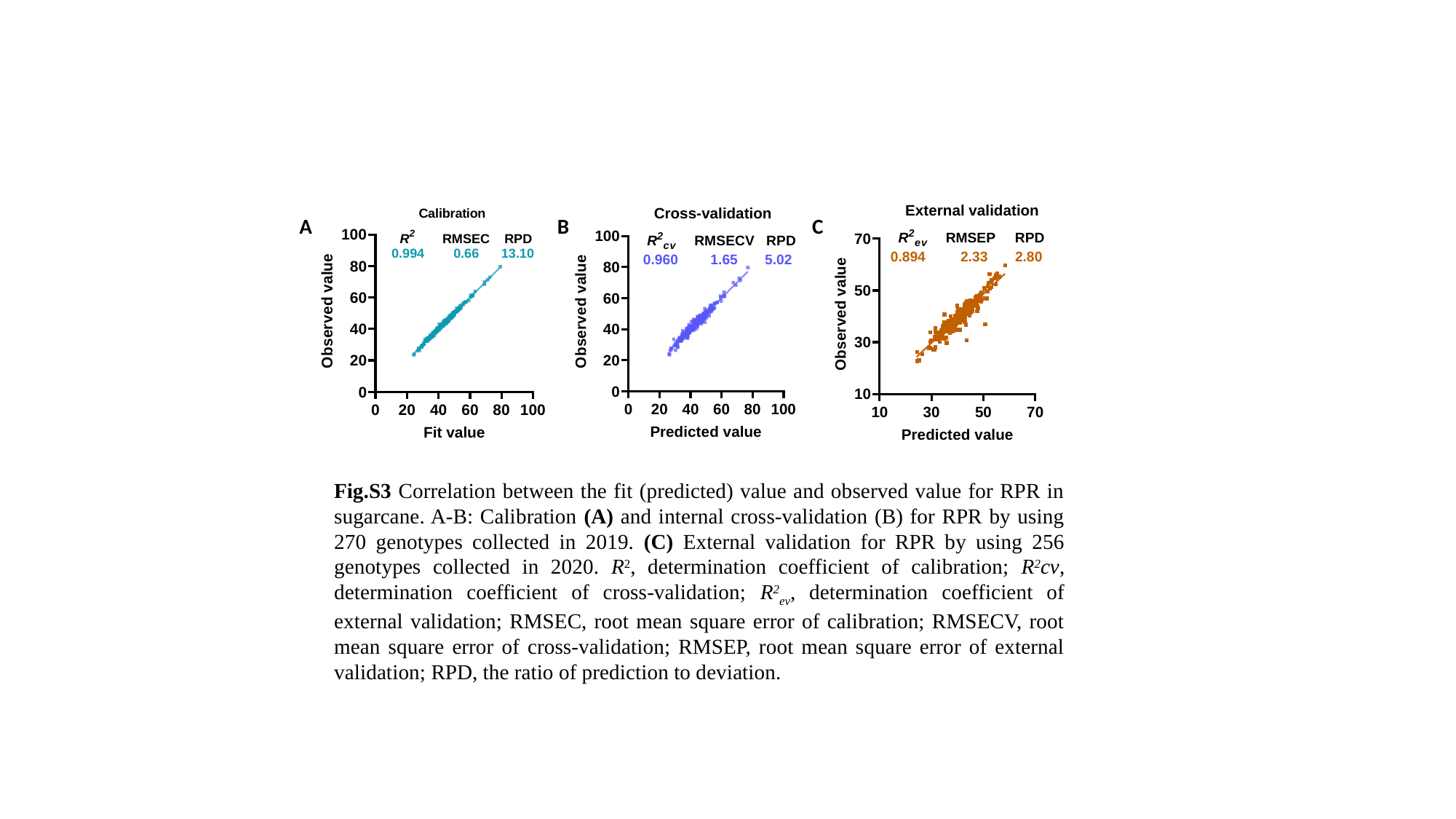

A
B
C
Fig.S3 Correlation between the fit (predicted) value and observed value for RPR in sugarcane. A-B: Calibration (A) and internal cross-validation (B) for RPR by using 270 genotypes collected in 2019. (C) External validation for RPR by using 256 genotypes collected in 2020. R2, determination coefficient of calibration; R2cv, determination coefficient of cross-validation; R2ev, determination coefficient of external validation; RMSEC, root mean square error of calibration; RMSECV, root mean square error of cross-validation; RMSEP, root mean square error of external validation; RPD, the ratio of prediction to deviation.

## Slide 5
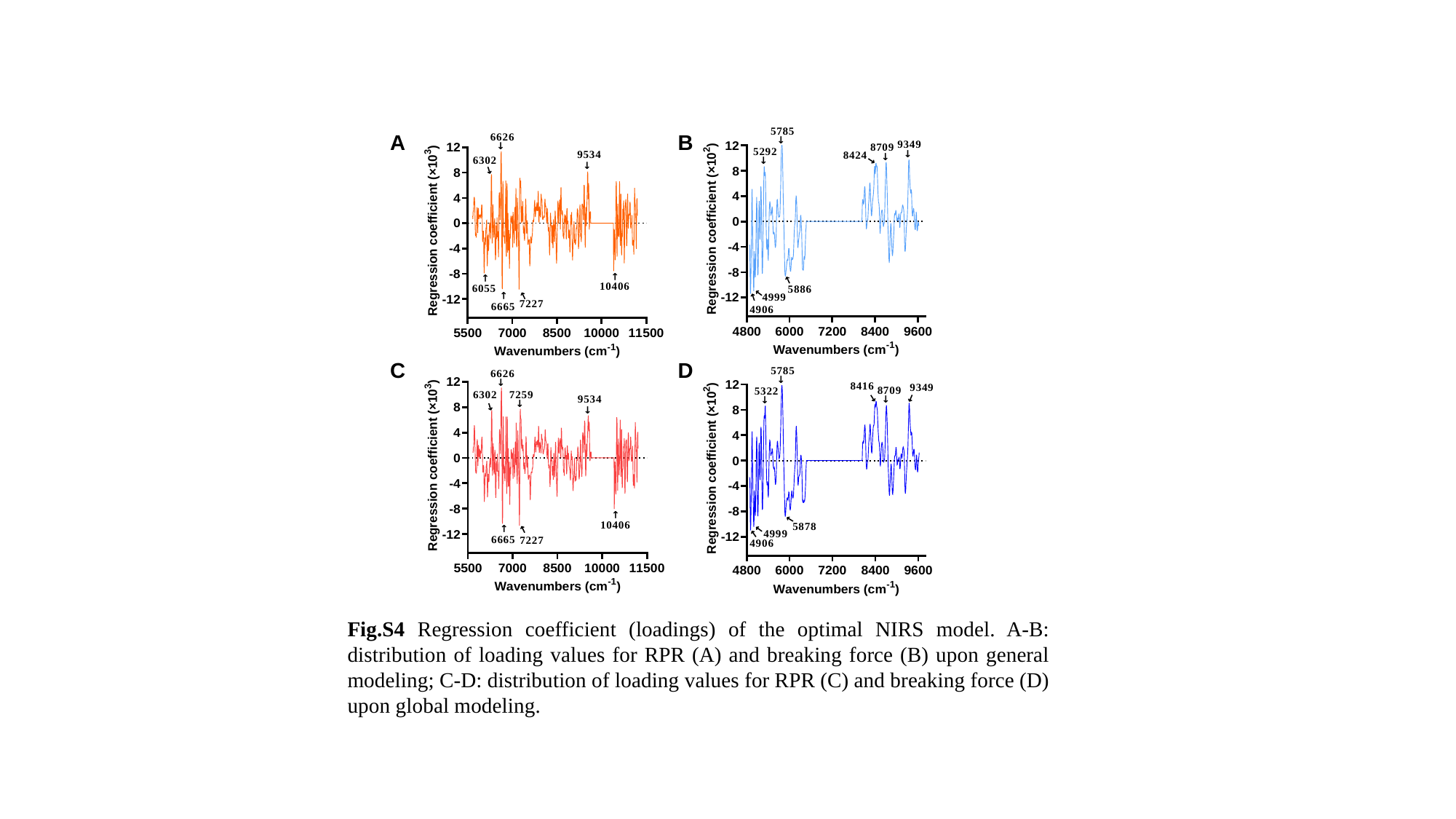

A
B
C
D
Fig.S4 Regression coefficient (loadings) of the optimal NIRS model. A-B: distribution of loading values for RPR (A) and breaking force (B) upon general modeling; C-D: distribution of loading values for RPR (C) and breaking force (D) upon global modeling.

## Slide 6
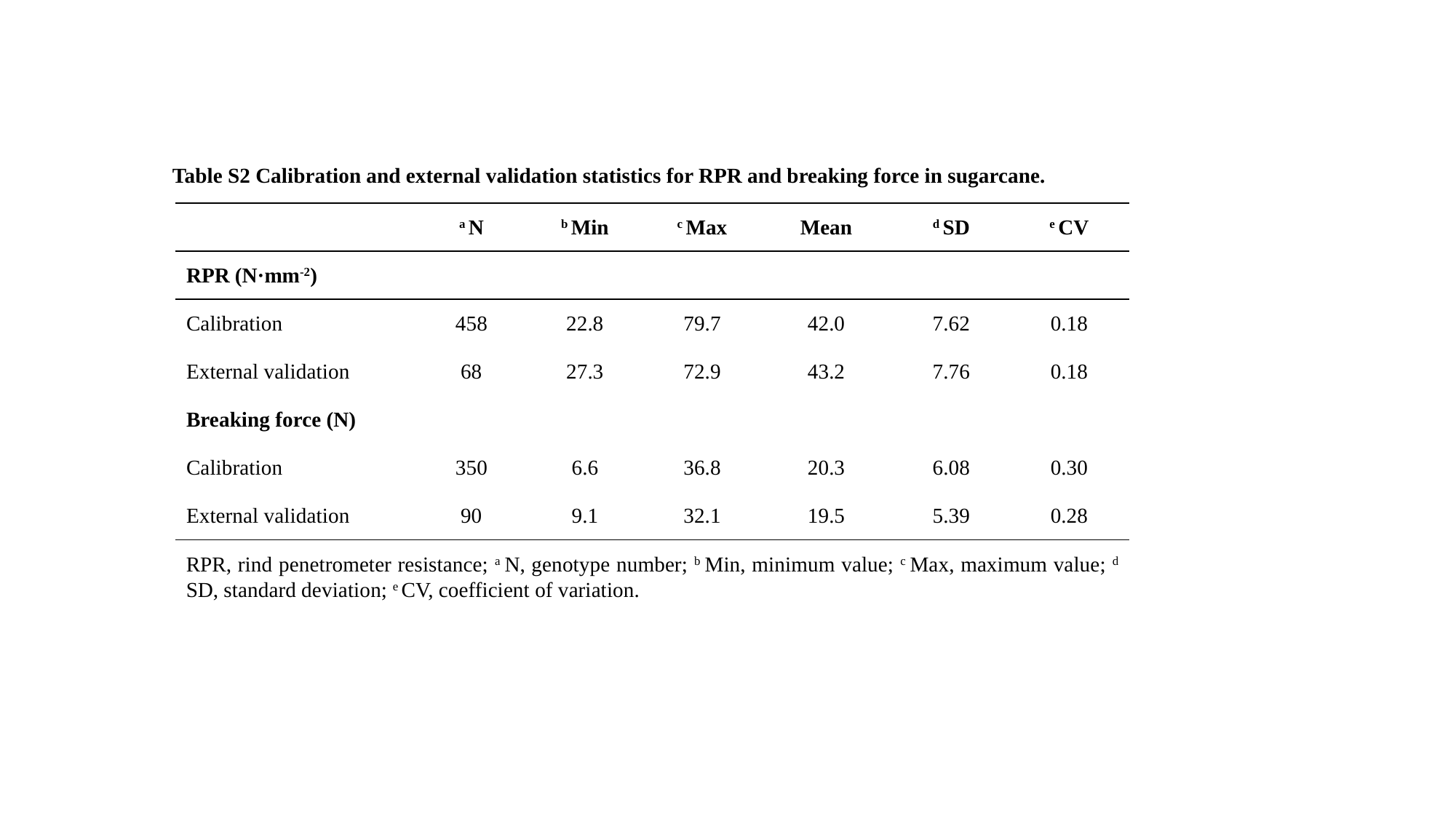

Table S2 Calibration and external validation statistics for RPR and breaking force in sugarcane.
| | a N | b Min | c Max | Mean | d SD | e CV |
| --- | --- | --- | --- | --- | --- | --- |
| RPR (N·mm-2) | | | | | | |
| Calibration | 458 | 22.8 | 79.7 | 42.0 | 7.62 | 0.18 |
| External validation | 68 | 27.3 | 72.9 | 43.2 | 7.76 | 0.18 |
| Breaking force (N) | | | | | | |
| Calibration | 350 | 6.6 | 36.8 | 20.3 | 6.08 | 0.30 |
| External validation | 90 | 9.1 | 32.1 | 19.5 | 5.39 | 0.28 |
RPR, rind penetrometer resistance; a N, genotype number; b Min, minimum value; c Max, maximum value; d SD, standard deviation; e CV, coefficient of variation.

## Slide 7
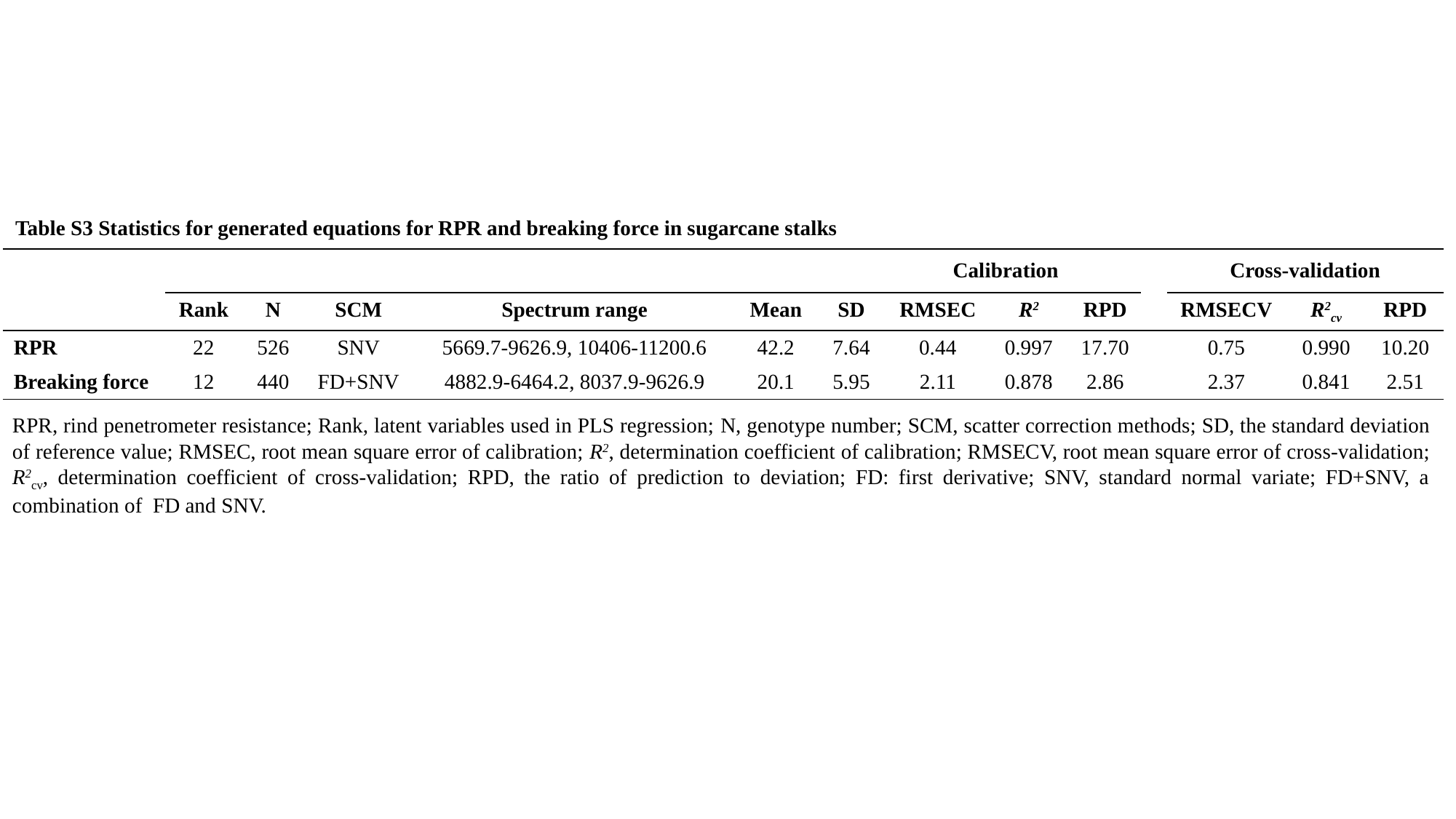

Table S3 Statistics for generated equations for RPR and breaking force in sugarcane stalks
| | Calibration | | | | | | | | | | Cross-validation | | |
| --- | --- | --- | --- | --- | --- | --- | --- | --- | --- | --- | --- | --- | --- |
| | Rank | N | SCM | Spectrum range | Mean | SD | RMSEC | R2 | RPD | | RMSECV | R2cv | RPD |
| RPR | 22 | 526 | SNV | 5669.7-9626.9, 10406-11200.6 | 42.2 | 7.64 | 0.44 | 0.997 | 17.70 | | 0.75 | 0.990 | 10.20 |
| Breaking force | 12 | 440 | FD+SNV | 4882.9-6464.2, 8037.9-9626.9 | 20.1 | 5.95 | 2.11 | 0.878 | 2.86 | | 2.37 | 0.841 | 2.51 |
RPR, rind penetrometer resistance; Rank, latent variables used in PLS regression; N, genotype number; SCM, scatter correction methods; SD, the standard deviation of reference value; RMSEC, root mean square error of calibration; R2, determination coefficient of calibration; RMSECV, root mean square error of cross-validation; R2cv, determination coefficient of cross-validation; RPD, the ratio of prediction to deviation; FD: first derivative; SNV, standard normal variate; FD+SNV, a combination of FD and SNV.
